# Multiple Roles of *Staphylococcus aureus* Enterotoxins: Pathogenicity, Superantigenic Activity, and Correlation to Antibiotic Resistance

**DOI:** 10.3390/toxins2082117

**Published:** 2010-08-10

**Authors:** Elena Ortega, Hikmate Abriouel, Rosario Lucas, Antonio Gálvez

**Affiliations:** Area de Microbiología, Departamento de Ciencias de la Salud, Facultad de Ciencias Experimentales, Universidad de Jaén, 23071-Jaén, Spain; Email: eortega@ujaen.es (E.O.); hikmate@ujaen.es (H.A.); rlucas@ujaen.es (R.L.)

**Keywords:** enterotoxins, *Staphylococcus aureus*, superantigens, MRSA, immune response

## Abstract

Heat-stable enterotoxins are the most notable virulence factors associated with *Staphylococcus aureus*, a common pathogen associated with serious community and hospital acquired diseases. Staphylococcal enterotoxins (SEs) cause toxic shock-like syndromes and have been implicated in food poisoning. But SEs also act as superantigens that stimulate T-cell proliferation, and a high correlation between these activities has been detected. Most of the nosocomial *S. aureus* infections are caused by methicillin-resistant *S. aureus* (MRSA) strains, and those resistant to quinolones or multiresistant to other antibiotics are emerging, leaving a limited choice for their control. This review focuses on these diverse roles of SE, their possible correlations and the influence in disease progression and therapy.

## 1. Introduction

*Staphylococcus aureus* is a common pathogen associated with serious community and hospital acquired diseases and has for a long time been considered as a major problem of Public Health [1]. Furthermore, *S. aureus* is often present in foods [2,3] and it is among the leading causes of foodborne bacterial intoxications worldwide [4,5,6]. Several virulence factors implicated in the pathogenesis of *S. aureus* strains have been described previously such as thermonuclease, hyaluronidase, lipases and hemolysisn [7,8,9], which are involved in tissue invasion of the host. Perhaps the most notable virulence factors associated with this microorganism are the heat-stable enterotoxins that cause the sporadic food-poisoning syndrome or foodborne outbreaks [10,11].

Staphylococcal enterotoxins (SEs) belong to a large family of staphylococcal and streptococcal pyrogenic exotoxins (PT), sharing common phylogenetic relationships, structure, function, and sequence homology. These toxins cause toxic shock-like syndromes and have been implicated in food poisoning and several allergic and autoimmune diseases. Included within this group are the staphylococcal enterotoxins, two forms of toxic shock syndrome toxin (TSST), and a group of streptococci pyrogenic exotoxins [12].

SEs function not only as potent gastrointestinal toxins but also as superantigens that stimulate non‑specific T-cell proliferation. Although these are two separate functions localized on separate domains of the proteins, there is a high correlation between these activities and in most cases a loss of superantigen activity (because of a genetic mutation) results in loss of enterotoxic activity as well [13].

Antimicrobial resistance is also an important public health concern worldwide. The development of resistance both in human and animal bacterial pathogens has been associated with the extensive therapeutic use of antimicrobials or with their administration as growth promoters in food animal production [14,15]. Apart from having pathogenic versatility, *S. aureus* can adapt rapidly to the selective pressure of antibiotics, with the emergence and spread of methicillin-resistant *S. aureus* (MRSA) isolates being a relevant example. MRSA was first described in 1961, the year in which methicillin was marketed [16], and actually most of the nosocomial *S. aureus* infections are caused by methicillin-resistant *S. aureus* strains [17], which have become a widely recognized cause of morbidity and mortality throughout the world [1,18,19]. *S. aureus* becomes methicillin resistant by the acquisition of the *mecA* gene which encodes a penicillin binding protein (PBP2a) with a low affinity for β-lactams. The strains producing PBP2a are resistant to all β-lactams [20]. Thus, MRSA strains resistant to quinolones or multiresistant to other antibiotics have been emerging, leaving a limited choice for their control [21,22]. 

## 2. Diversity of *Staphylococcus aureus* Enterotoxins

The first well-documented report, which clearly identified a *Staphylococcus aureus* toxin as the cause of food poisoning outbreaks, was done by Dack *et al.* [23]. They isolated a pigment-forming *Staphylococcus* present in large numbers in a Christmas cake responsible for a food poisoning incident, and sterile filtrate from a broth, in which the organism was grown, produced illness when ingested by human volunteers. Initially, five antigenic variants of *S. aureus* enterotoxins designated SEA through SEE were identified and reported in the literature [24,25,26,27,28]. Since then, new variants have been identified and designated SEH to SElR, and SElU in the order that they were discovered [29]. These staphylococcal superantigenic toxins can be divided into three large groups and one minor group on the basis of similarity of amino acid sequences. Most toxins of the three groups, including staphylococcal enterotoxins A and B (SEA and SEB), exhibit strong emetic activity in primates; toxic shock syndrome toxin-1, grouped as the minor group, does not possess emetic activity in primates. It is noteworthy that toxins designated SE-like toxins, such as SElP, which either have not been examined for emetic activity or have been reported not to have emetic activity, have been discovered in *S. aureus* strains [30]. Recently, two novel staphylococcal enterotoxins, types S and T have been identified and characterized, and SElR has been shown to possess emetic properties, so it should be designated SER from now on [31].

Enterotoxins are short secreted proteins, soluble in water and saline solutions. They share common biochemical and structural properties and are remarkably resistant to heat: the potency of these toxins can only be gradually decreased by prolonged boiling or autoclaving. Excepting toxic shock syndrome toxin-1 (TSST-1), they are highly stable and resistant to most proteolytic enzymes, and thus retain their activity in the digestive tract after ingestion [32]. Their biological properties include induction of high fever similar to bacterial endotoxin induction, lethal shock in animals resulting from excessive intravenous doses, enhanced host susceptibility to endotoxin lethality, cytokine production, and polyclonal T-lymphocyte proliferation as seen with superantigens (SAgs)[33,34].

As to the SE production and regulation, *S. aureus* has developed overlapping subsets of accessory genes dedicated to regulating the expression of other genes, including SEs and TSST-1.The most well‑known and predominant regulon in *S. aureus* is the *agr* system (accessory gene regulator). It is a quorum-sensing system activated at high cell density. *In vitro*, upon activation, *agr* downregulates the expression of genes encoding surface proteins and upregulates genes encoding exoproteins, such as SEB, SEC, SED and TSST-1 [35,36]. However, this is not the global rule for SE expression because SEA is constitutively expressed by *S. aureus* while SEG and SEI were initially thought to be produced only at low bacterial concentrations [37]. Until now the question of SEG and SEI production seems to be controversial, as there is no evidence confirming this [38,39]. Indirect evidence indicates only that its expression can be triggered by environmental factors [40,41]. 

Arginine and cystine are also necessary for both growth and SE production in five strains of *S. aureus* that produce SEA, SEB or SEC, while the necessities for other aminoacids vary with the strain [42]. On the contrary, glucose has been shown to have an inhibitory effect on SE production, especially for SEB and SEC [43]. This inhibitory effect has been attributed to a drop in pH, as a consequence of glucose metabolism. These observations could also be correlated with *agr*-dependent synthesis of these SEs, as glucose and low pH indeed have an inhibitory effect on *agr*-expression [35].

## 3. Pathogenicity of SE

*Staphylococcus aureus* is an unusually successful and adaptive human pathogen that can cause epidemics of invasive disease despite its frequent carriage as a commensal. Over the past 100 years and more, *S. aureus* has caused cycles of outbreaks in hospitals and the community and has developed resistance to every antibiotic used against it, yet the exact mechanisms leading to epidemics of virulent disease are not fully understood. This microorganism is a versatile pathogen capable of causing a wide range of human diseases due to a combination of toxin-mediated virulence, invasiveness, and antibiotic resistance. The spectrum of staphylococcal infections ranges from pimples and furuncles to toxic shock syndrome and sepsis, most of which depend on numerous virulence factors. On the other hand, some infections, such as staphylococcal food poisoning, rely on one single type of virulence factor: the SEs. 

### 3.1. Staphylococcal Food Poisoning

Many different foods can be a good growth medium for *S. aureus*, and have been implicated in staphylococcal food poisoning, including milk and cream, cream-filled pastries, butter, ham, cheeses, sausages, canned meat, salads, cooked meals and sandwich fillings. The origins of staphylococcal food poisoning differ widely among countries and this may be due to differences in the consumption and food habits in each of the countries. In any case, the main sources of contamination are humans (handlers contaminate food via manual contact or via the respiratory tract by coughing and sneezing), and contamination occurs after heat treatment of the food. Nevertheless, in food such as raw meat, sausages, raw milk, and raw milk cheese, contaminations from animal origins are more frequent and due to animal carriage or to infections (e.g., mastitis)[44].

The symptoms of staphylococcal food poisoning are abdominal cramps, nausea, vomiting, sometimes followed by diarrhea after a short period of incubation [44]. In contrast to the well-known clinical manifestations, the physiopathology of the symptoms is only partially understood. Gastroscopic examination of patients with acute SE intoxication show gastrointestinal damage with patchy mucosal hyperemia, regional edema, muscular irritation, erosion, petechiae and purulent exudates [45]. In these areas, an influx of polymorphonuclear cells into the lamina propria and epithelium can be observed. SEs can penetrate the gut lining and gain access to both local and systemic immune tissues. It has been postulated that local immune system activation by SEs could be responsible for the gastrointestinal damage observed as well as mast cell activation. The release of inflammatory mediators (histamine and leukotriene) and neuroenteric peptide substance P upon SE activation could also be responsible for the gastrointestinal tract damage [46]. Histamine release is also associated with other types of food poisoning, but the question still remains as to whether synthesis of these mediators is induced directly or indirectly by SEs. Diarrhea in staphylococcal food poisoning may be due to the inhibition of water and electrolyte reabsorption in the small intestine by SEs as observed both *in vitro* and in an animal model. Emetic activities of SEs and related toxins are not equivalent. TSST-1 is not emetic, probably due to its sensitivity to digestive tract enzymes. Other SEs such as SEI and SElL are either weak or not emetic; these two toxins lack the disulfide bond characteristically at the top of domain B, which may stabilize a crucial conformation within or adjacent to the loop probably important for emesis activity [47]. Studies involving mutations in various SEs show no link between reduction on T cell proliferation and emesis [13,48], although studies that dissociate monocyte activation from emesis are still lacking. 

### 3.2. Toxic Shock Syndrome (TSS)

First reported by Todd [49], TSS is a major systemic illness associated with acute intoxication with TSST-1 and SEs. It is characterized by an acute onset of high fever, diffuse erythematous rash, desquamation of the skin, hypotension, and the involvement of three or more organ system failures. The TSS pathophysiology model involves a capillary leak syndrome stemming from toxin- and cytokine-mediated endothelium damage. *In vitro*, SAgs as well as proinflammatory cytokines are lethal for endothelial cells [50]. Regarding the respective roles of monocytes and T lymphocytes in the host inflammatory response to SAgs, the literature remains controversial. Dinges *et al.* [51] showed T-independent mortality induced by TSST-1 in a rabbit model. SEB induced the production of TNF‑α by isolated monocytes but not by nonmonocytes or purified T cells, suggesting that the proinflammatory reaction can occur without the presence of T cells. On monocytes, SAgs interact mainly with MHC class II. SEA, SED and SEE possess a cysteine domain involved in high-affinity binding to MHC class II that is absent in SEB, SEC and TSST-1 [52], so it has been hypothesized that differences in SAgs inflammatory properties could be a reflection of respective differences in their MHC class II binding properties. Wright and Chapes [53] proposed another mechanism of action for SAgs on monocytes. They have demonstrated that SEA could also interact with MHC class I on monocytic cells deficient for MHC class II and induce an important cytokine response, most notably the release of TNF-α and IL-6. On the other hand, several reports proposed a predominant role for T-lymphocytes in inducing the inflammatory response. Marrack *et al.* [54] showed that the pathological effect of SEB may be the consequence of massive T cell stimulation. Lastly, it has been suggested that the synergy between Gram-negative endotoxins and SAgs contributes to TSS lethality through TNF, as *in vitro* SAg-MHC II interaction and IFN-γ secreted by SAg-activated T cells induced an increase in the expression of TLR4, the cell receptor of LPS [55]. In the same way, LPS enhanced MHC II expression on APC [56].

### 3.3. Noninfectious Disease

Several reports have described Vβ-specific enrichments of T cells in different diseases, such as Kawasaki´s disease and autoimmune diseases, so it has been proposed that SAgs might also contribute to the pathogenesis of noninfectious diseases through activation of T-cells specific for self-antigens. Kawasaki´s disease is an acute febrile disease in children that resembles TSS, although the etiological agent remains unknown [57]. Most of the patients with this disease are colonized with *S. aureus* carrying the *tst* gene and exhibit selective expansion of a Vβ2 subset of T cells. Moreover, some patients increase their levels of IgM antibodies against SAg in the weeks following diagnosis with KD [58,59]. It has been also reported a selective expansion of Vβ subsets of synovial T cells in arthritis [60] and in the pancreas of insulin-dependent diabetes mellitus [61]. Recently, it has also been postulated that SAgs could play a role in skin and airway allergies, such as atopic dermatitis, as IgE antibodies directed against SEA and SEB were detectable in these patients, and their levels were associated with the disease, suggesting that SAgs could function as conventional allergens [62].

## 4. Enterotoxin’s Superantigenic Activity

The term superantigen was coined by John Kappler in 1989 [63] to denote the ability to stimulate large numbers of T cells, although this was an immunologist’s redefinition of a group of toxins that had been studied for many years prior as staphylococcal enterotoxins (SE) that caused food poisoning and toxic shock and the streptococcal pyrogenic exotoxins (SPEs), agents causing scarlet fever, pyrogenicity, and streptococcal TSS (STSS). Potent *in vitro* lymphocyte proliferation was a common feature of all the purified SEs, SPEs, and TSST-1. In a series of independent studies, the SE and SPEs were found to share two common functions: the ability to bind as whole molecules directly to MHC class II [64,65] and also to the TCR β-chain [66] outside the normal antigen-binding interface that governed peptide MHC recognition [67]. Charles Janeway [68] first suggested that the SEs and the determinants encoded by the *Mls* (Mouse lymphocyte stimulating) system might act by cross-linking the MHC class II and TCR Vβ domain. Perhaps the most significant discovery in recent years has been the finding that SAgs belong to a much larger family that includes the staphylococcal superantigen-like proteins (SSLs). These are built from the same two polypeptide domains but display a different set of anti-immune functions targeting the innate response. Together the SAgs and the SSLs reveal how a simple protein has been repeatedly modified to produce an armamentarium of immune defense molecules.

At present, 20 serologically distinct staphylococcal SAgs have been described, comprising TSS toxin-1 (TSST-1), the SEs A-E, G-J, and the staphylococcal enterotoxin-like toxins (SEl) K-R and U, and the recently identified SEl-U2 and SEl-V [69]. Not all SAgs are enterotoxic and a new nomenclature was introduced in 2004 to distinguish those with proven emetic activity (SEs) with those that remain unconfirmed (SEls) [30].

All the staphylococcal SAgs are potent T cells mitogens but exhibit different preferences for MHC class II alleles and also produce distinct TCRVβ profiles. This suggests that their diversity has been driven by a need to stimulate as many different T cells as possible while retaining MHC and TCR as target molecules. Superantigens, as well as the other extracellular staphylococcal virulence factors, are accessory proteins, *i.e* they are not required for growth and multiplication of the organism, and are often encoded by accessory genetic elements such as prophages, transposons, plasmids, and pathogenicity islands (PIs). These mobile elements are not uniformly distributed among clinical isolates and there are significant variations in these regions between different strains, most likely due to horizontal transfer [70].

The three dimensional structures of 10 staphylococcal and seven streptococcal SAgs have been solved by crystallography. All the structures reveal compact two domain globular proteins that range in size from 20 to 28 kDa. The N-terminal extension contributes substantially to the TCR-binding site that is formed as a shallow depression between the two protein domains. The SAgs are noted for their impressive stability to denaturing conditions such as heat and acid and as a consequence are not completely destroyed by mild cooking of food, hence their potency in food poisoning.

The notion that SAgs simultaneously bind MHC class II and TCR is unrealistic. The superantigen must first bind to MHC class II to concentrate onto the surface of the APC, making use of the adhesion and accessory molecules that form the immunological synapse. At some early point after APC binding, the surface concentration is sufficient to successively engage and cross-link multiple TCR molecules, resulting in strong TCR signaling, activation, and rapid cytokine production. The affinity of SAgs towards MHC class II molecules is thus typically 10 to 100-fold higher than its affinities towards TCR. Those SAg/TCR solution interactions that have been quantified typically exhibit the same affinity and binding rates as those measured for TCR/peptide MHC interactions [71]. Based on crystallographic data of SAgs complexed with MHC class II, the interaction of SAgs with MHC class II can be classified into 4 groups: (i) binding to MHC class II α-chain entirely peripheral to the bound antigen peptide (peptide‑independent binding), e.g., HLA-DR/SEB [72]; (ii) binding to MHC class II α-chain and extension over the bound peptide (peptide-dependent binding), e.g., HLA-DR/TSST-1 [73]; (iii) zinc-mediated binding to MHC class II β-chain and extension over the bound peptide (peptide-dependent binding), e.g., HLA-DR2/SPEC [74], HLA-DR1/SEH [75], HLA-DR1/SEI [76]; and (iv) SAgs that combine both (i) and (ii) binding modes such as SEA to cross-link MHC class II [77,78].

On the other hand, SAgs interact with TCR molecules by binding primarily to the variable region of the β-chain (Vβ-domain), resulting in oligoclonal stimulation of a defined T-cell repertoire, potentially activating more than 20% of all T cells. Crystal structures of SAgs in complex with Vβ domains have revealed structurally diverse interactions, although binding to the TCRVβ CDR2 loop seems to be a requirement for all bacterial SAgs, whereas binding to other Vβ domain regions appears to result in Vβ domain specificity and crossreactivity of SAgs [71].

Attempts to separate the enterotoxin activity from the SAg activity have yielded inconclusive results. Nevertheless, a high correlation exists between these activities since, in most cases, genetic mutation resulting in a loss of superantigen activity results also in a loss of emetic activity. A highly flexible disulfide loop within the N-terminal domain has been implicated with the emetic activity but does not appear to be an absolute requirement [43]. It has also been suggested that the production of monocyte chemoattractant protein-1 (MCP-1) by intestinal myofibroblasts (IMFs) might play a role in SEA food poisoning [79,80]. Cultured human IMFs were able to bind SEA in an MHC class II-dependent fashion, resulting in increased release of MCP-1 and the consequential production of IL-6 and IL-8. However, this mechanism fails to explain the emetic activity of other SEs, as it depended on the cross-linking of MHC class II molecules on IMFs by SEA, whereas incubation with SEB did not trigger a response. Hu *et al.* [81] postulated SEA-induced emesis through rapid serotonin release and showed in an animal model that SEA induces 5-hydroxytryptamine (5-HT) in the intestine, rather than in the brain. Receptors on vagal afferent neurons are essential for SEA-triggered emesis.

The most likely role of SAgs is during the early stages of infection when the very low levels of SAg activate only local T cells. At this stage, the host response to *S. aureus* or *S. pyogenes* infection is entirely dependent on myeloid cells such as neutrophils and macrophages that are rapidly drawn to and engulf the invading bacteria, triggered by inflammatory mediators and Fc receptor and complement-mediated mechanisms. One possible advantage of T-cell activation at the infection site might be to produce cytokines such as IL-2, IFN-γ, and TNF-α that suppress local inflammation. 

## 5. Antibiotic Resistance in *S. aureus* Correlation to Enterotoxigenic Strains

Food is an important factor for the transfer of antibiotic resistances. Such transfer can occur by means of antibiotic residues in food, through the transfer of resistant food-borne pathogens or through the ingestion of resistant strains of the original food microflora and resistance transfer to pathogenic microorganisms [1,82]. *S. aureus* strains are known to be frequently resistant to antibiotic therapy due to their capacity to produce an exopolysaccharide barrier and because of their location within microabscesses, which limit the action of drugs [83].

As previously related, it is well known that determinants of resistance to antibiotics and other toxic substances in staphylococci, as in other pathogens, are largely carried by accessory genetic elements, especially plasmids, transposons and their relatives. A particularly important resistance determinant in staphylococci is that for methicillin and other β−lactamase resistant β-lactam compounds, giving rise to the notorious MRSA acronym. Although it has been clear for some time that a novel penicillin-binding protein, PBP2a [84,85], is responsible for this resistance, and that the PBP2a gene is not native to *S. aureus*, there has been considerable uncertainty regarding the nature of the accessory genetic elements that carry it. These were thought at one time to be plasmids or transposons [86], but have recently been shown definitively to be a family of large chromosomal insertions that belong to the general category of chromosomal islands [87] and are appropriately regarded as resistance islands.

The staphylococcal pathogenicity islands (SaPIs) and staphylococcal chromosome cassette methicillin-resistance islands (SCC*mecs*) represent two well-defined classes of novel mobile elements that are presumably responsible for the horizontal transmission of SAg and certain resistance determinants. It seems remarkable that the SaPIs carry only SAg determinants and the SCC*mecs* carry only resistance determinants.

Several reports have recently analyzed the prevalence of enterotoxin genes in methicillin-resistant and methicillin-susceptible (MSSA) isolates of *S. aureus*. Sila *et al.* [88] found 7 genes more frequently detected in MRSA isolates: *sea*, *seb*, *sed*, *seg*, *sei*, *sej* and *eta*, coding for the production of enterotoxins A, B, D, G, I, J and the exfoliative toxin A. On the other hand, the *pvl*, *tst* and *sec* genes for Panton-Valentine leukocidin, TSST-1 and enterotoxin C were most frequent in MSSA. Statistical analysis of the comparison of the prevalence of virulence factors in the two studied groups showed significant difference in detection of the *seg* and *sei* genes. The characterization by Pereira *et al.* [17] of different *S. aureus* isolates collected from different food origins showed that 38% of the studied strains were MRSA, and 19% of the tested isolates were both enterotoxigenic and oxacillin positives. A correlation was observed between the antibiotic resistance profile of the strains isolated from cases of bovine mastitis and from the raw cow’s milk; in general, these specific isolates were demonstrated to be resistant to penicillin and ampicillin and more sensitive to the other antibiotics possibly as a result of treating mastitis cases with β-lactams. In the case of isolates from fermented sausages, a higher overall resistance to antibiotics was observed (again possibly from using antibiotics as growth promoters in animal feed). Normanno *et al.* [89] studied 160 *S. aureus* strains from food of animal origin products in Italy and six (3.75%) harbored the *mecA* gene. Each *mecA* positive strain was derived from a different food sample. In particular, four strains were from bovine milk, one strain from mozzarella cheese, and one from pecorino cheese. All the MRSA strains synthesized SEs. Two strains synthesized SEA and SED (33.3%), one strain SEC and SED (16.6%), two strains SED (33.3%) and one strain SEC (16.6%). Three MRSA strains (50%) belonged to the non-host-specific (NHS) biovar and three strains (50%) belonged to the ovine biovar. However, the role and the source of food contamination are still unclear since only few reports on the presence and possible origin of MRSA in foods are available. In his analysis of 1913 specimens from food-producing animals, including milk and meat of beef, pig and chicken origin, Lee [90] found 15 strains harboring the *mecA* gene. Most of the MRSA isolates were from milk and three from chicken. Lee concluded that contaminated foods of animal origin may represent a source of MRSA infection for humans. Recently, out of 444 samples of raw chicken meat examined by Kitai *et al.* [91], two (0.45%) harbored MRSA strains. One of the two strains isolated (one per positive sample) synthesized SEC and was thus capable of causing foodborne illness. Interestingly, these two MRSA strains belonged to the human biovar, suggesting that food handlers had been the source of contamination. 

Until now the correlation of antibiotic resistance and enterotoxigenicity of *S. aureus* has not been clearly supported by published results showing that food poisoning caused by MRSA is different of that caused by MSSA, except for the difference in prevalence of SE genes in these two populations. In turn the role of antibiotic resistance in the pathogenicity of SE-producing *S. aureus* has been revealed under specific conditions, e.g., such as those leading to AAD (antibiotic associated diarrhea) development. Alteration of gut flora by antibiotic therapy seems to be important in regard to the expression of pathogenic properties of intestinal MRSA, frequently the only bacteria able to survive the presence of antimicrobials. Clinical parameters are higher in ADD patients colonized with enterotoxigenic MRSA than in those suffering from diarrhea not associated with MRSA or colonized with non-enterotoxigenic MRSA [92]. 

## 6. Conclusions

*Staphylococcus aureus* enterotoxins function both as potent gastrointestinal toxins as well as superantigens that stimulate non-specific T-cell proliferation. Structurally, these two functions are located in two separate domains, so were initially considered to target different host tissues. An open question remains whether these separate functions are related. In recent years, staphylococcal enterotoxins with superantigenic activities have drawn more and more attention because of their anti‑infection and anti-tumor therapy potential. Superantigenic toxins with trace-level could significantly stimulate the immune system in primates and rodents resulting in massive cytokine release. To decrease the toxicity and enhance the superantigenic activities, some mutants of staphylococcal enterotoxins such as mSEA and mSEC2 have been constructed for tumor or infection therapy purposes.

This question may also be related to how the toxins enter the body via the intestine. It has been found that epithelial cells are capable of a dose-dependent, facilitated transcytosis of SEs (tested on SEB and TSST-1) and that ingested SEB for example appears in the blood more readily than SEA. A working hypothesis is that the enterogenic activity may facilitate transcytosis, thus enabling the toxin to interact with T cells in the bloodstream, leading to superantigenic activity. More studies are needed to answer the questions regarding the activities of SEs as enterogenic and as superantigens. Moreover, understanding the mechanisms of subversion of the immune response by *S. aureus* will not only lead to new insights into the pathophysiology of infection and to new antimicrobial strategies, but could also provide the platform for the design of new therapies in inflammatory and autoimmune disorders.

*S. aureus* is also well established as a clinical and epidemiological pathogen and the potentially pathogenic role of *S. aureus* as a food-borne pathogen should not be neglected. Antibiotic-resistant isolates might be transmitted to humans by the consumption of food products containing such resistant and multiresistant bacteria and the use of antibiotics as growth promoters in animal husbandry, especially of those commonly used for both human and animal care, should be avoided.

Recent findings highlight the high potential risk for consumers in the absence of strict hygienic and preventative measures to avoid the presence of *S. aureus* isolates and SEs production in foods, emphasizing the need for improved hygiene practices during food processing and also during the distribution and consumption of the final food products.
